# EXAMINING THE INTERPERSONAL-PSYCHOLOGICAL THEORY OF SUICIDE AMONG OLDER KOREANS: DOES AGE MATTER?

**DOI:** 10.1093/geroni/igae098.3060

**Published:** 2024-12-31

**Authors:** Jiyoung Lyu, Hye-Mi Kim, Keung-Ja Hong, Byoung-Jun Park

**Affiliations:** Hallym University, Chuncheon, Kangwon-do, Republic of Korea; Hallym University, Chuncheon, Kangwon-do, Republic of Korea; Sogang University, Seoul, Republic of Korea; Sogang University, Seoul, Republic of Korea

## Abstract

The interpersonal–psychological theory of suicide proposes that perceived burdensomeness, thwarted belongingness, and the acquired capability for suicide leads to suicide attempts. However, whether this applies to all age groups is not clear. Therefore, this study was aimed to apply the interpersonal-psychological theory of suicide to older Koreans of different age groups. Participants of this study were 1,200 older adults aged 65 years and above (347 Young-old, 853 Old-old) who completed 2020 surveys on Elderly Life Conditions in Chuncheon, Korea. The Interpersonal Needs Questionnaire, the Suicidal Ideation Scale, and the Acquired Capability for Suicide Scale were used to assess participants’ perceived burdensomeness, thwarted belongingness, suicide attempts, and the capability for suicide. A multivariate regression results showed that thwarted belongingness was significantly associated with suicide attempts among young-old group, while perceived burdensomeness was significantly associated with suicide attempts among old-old group. These results suggest that the mechanisms around suicide attempts among older Koreans may be different by different age groups.

